# Getting Ahead of the Arms Race: Hothousing the Coevolution of VirusTotal with a Packer

**DOI:** 10.3390/e23040395

**Published:** 2021-03-26

**Authors:** Héctor D. Menéndez, David Clark, Earl T. Barr

**Affiliations:** 1 Computer Science Department, Middlesex University London, London NW4 4BG, UK; 2 Computer Science Department, University College London, London WC1E 6BT, UK; david.clark@ucl.ac.uk (D.C.); e.barr@ucl.ac.uk (E.T.B.)

**Keywords:** coevolution, adversarial machine learning, malware arm race, EEE, VirusTotal, hothouse

## Abstract

Malware detection is in a coevolutionary arms race where the attackers and defenders are constantly seeking advantage. This arms race is asymmetric: detection is harder and more expensive than evasion. White hats must be conservative to avoid false positives when searching for malicious behaviour. We seek to redress this imbalance. Most of the time, black hats need only make incremental changes to evade them. On occasion, white hats make a disruptive move and find a new technique that forces black hats to work harder. Examples include system calls, signatures and machine learning. We present a method, called Hothouse, that combines simulation and search to accelerate the white hat’s ability to counter the black hat’s incremental moves, thereby forcing black hats to perform disruptive moves more often. To realise Hothouse, we evolve EEE, an entropy-based polymorphic packer for Windows executables. Playing the role of a black hat, EEE uses evolutionary computation to disrupt the creation of malware signatures. We enter EEE into the detection arms race with VirusTotal, the most prominent cloud service for running anti-virus tools on software. During our 6 month study, we continually improved EEE in response to VirusTotal, eventually learning a packer that produces packed malware whose evasiveness goes from an initial 51.8% median to 19.6%. We report both how well VirusTotal learns to detect EEE-packed binaries and how well VirusTotal forgets in order to reduce false positives. VirusTotal’s tools learn and forget fast, actually in about 3 days. We also show where VirusTotal focuses its detection efforts, by analysing EEE’s variants.

## 1. Introduction

The malware detection arms race favours black hats, who can effectively respond to detection efforts with low-cost incremental moves. White hats are trapped chasing black hats’ moves. To get ahead, white hats must make a disruptive move. A good example of such a disruptive move is forcing black hats to evade machine learning-based detectors. Once black hats have adapted to the new detection technique, the race reverts to a status quo where black hats need only make trivial modifications to a malware sample. This has already happened with malware detection, where black hats can easily and effectively add opaque predicates to evade machine learning algorithms [1,2]. Black hats are exploiting the new status quo with a flood of cheap, evasive variants and are provoking white hats to develop new disruptive techniques; white hats’ efforts currently center on semantics-based detection methods [3] and general improvements to their machine learning based detectors [4]. Simulating these black hats’ incremental moves will speed white hats’ ability to detect variants based on them, and force black hats to make disruptive moves instead of incremental ones. We study this arms race as a coevolution [5].

Search and, specifically, genetic algorithms simulate evolutionary behaviour, usually with a restricted palette so as to make the simulation tractable [6]. We use genetic algorithms to place evolutionary stress on state of the art malware detectors so that they have to improve their detection and quickly improve their ability to detect incrementally produced variants. This evolutionary stress ’hothouses’ the detector speeding its acquisition of detection power, modulo the last disruptive move (Section 2). The question that provoked this research is “Can we engineer a situation in which artificial evolutionary behaviour can assist in ‘hothousing’ the actual malware detection arms race to the benefit of the detection side?”. This necessitate coevolution. In this coevolution, the detector and the evader try to defeat each other.

VirusTotal’s [7] existence makes answering our question possible. VirusTotal is a framework where different vendors can contribute with anti-virus (AV) engines. At the time of this study there were 56. The plan was to treat the AV engines of VirusTotal as black boxes with which we interact in real time. We measured success in terms of driving down their median detection proportion across the malware submitted to anti-virus engines. Our long term aim is, of course, to strengthen them through coevolution. In addition, we studied their responses individually and in aggregate learn what the black boxes were doing. During the study, we polymorphically protect packed malware from entropy-based detection by adding controlled entropy regions, chosen and placed so as to make our packed binaries indistinguishable from benign-ware (Section 3). Subsequently, we added the ability to learn from the median detection rate to vary these regions so that the detection rate was lowered.

Our packer, called EEE [2] (the evolutionary packer or El Empaquetador Evolutivo in Spanish), focused on Windows malware because of the importance of the Windows’ ecosystem. Designing a tool to modify binary Windows malware and preserve behaviour and maintain viability, such as size, offered challenges. Polymorphic engines working at the machine code level with unpacking and repacking abilities were not available to us, so we created EEE by adding new capabilities to an open source packer, UPX. EEE creates variants by combining its packing abilities, encryption and the injection of controlled entropy regions. The packer learns how to combine these skills to disrupt the detector. UPX is able to repack PE and ELF binaries, among others, therefore it is able to manipulate almost any Windows malware in the wild. It has the advantage that it is widely used to pack benign programs so that it did not signal malware of and in itself. However, any packer is vulnerable to dynamic analysis and we needed to protect against that (Section 3).

**Figure 1:**
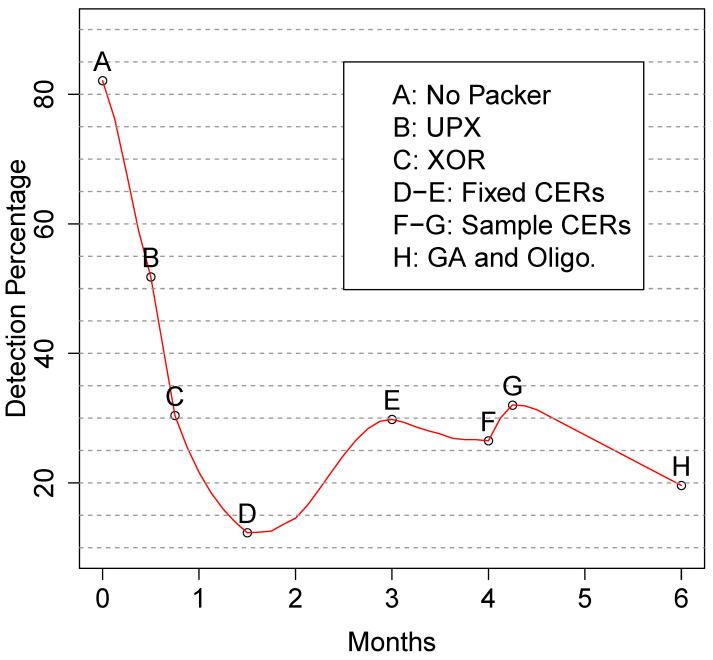
EEE coevolution with VirusTotal; delayed start and variable delimiters accompanied the addition of sampled CERs: interpolating from 07/16 to 01/17. The non-linear interpolation estimates the responds of VirusTotal during the coevolution.

Figure 1 tracks the coevolution of our versions of EEE and VirusTotal, in terms of malware detection. EEE has already shown its effectiveness by attacking machine learning detectors based on frequency features [2]. Here, we extend it to attack real anti-virus engines. We create different version of EEE including protections and skills. Its last version, which appears at the last point of Figure 1 (H) reduced VirusTotal’s median detection rate from 32.5% to 19.6%. The same figure also shows the VirusTotal AV engine’s ability to evolve in response at points E and G on the same graph. Point A (82.1%) in Figure 1 is the initial, median detection rate and the response to the novelty of the packer can be witnessed in the precipitous drop between points A and D (12.3%) on the graph as we added new capabilities to UPX (Section 4.1).

VirusTotal responded throughout our coevolution, at times surprisingly quickly. At one point, we observed the growth of false positives in its detections, which we experimentally confirmed (Section 4.2). Later, the AV engines involved began to correct the overcompensation (Section 4.3). Our current assessment is that the constituent AV engines have not yet learned to detect the results of the abilities of EEE directly but instead focus on its stub where critical information unique to EEE is stored. One of our experiments demonstrates this (Section 4.4). Another identifies four of the VirusTotal AV engines as champions at detecting us (Section 4.4). The paper presents the following contributions:

- We introduce a learning method that can show white hats where they are more vulnerable, to help them to anticipate black hats (Section 2).
- We instantiate this framework for information theoretic malware detection by evolving a polymorphic engine, EEE, and coevolve it against VirusTotal (Section 3).
- We demonstrate that artificial evolution via a genetic algorithm can drive down the median detection rate of state of the art malware detectors in the context of a coevolutionary detection arms race (Section 4).

## 2. Hothousing the Coevolution

Like any arms race, malware has a coevolutionary aspect. As we already observed, the arms race favours the black hats who are evading. This is similar in other arm races in cybersecurity. Obfuscation and de-obfuscation [8], spam and filters [9] and network neutrality [10] are all examples. Arms races proceed through series of breakthroughs, each conferring an advantage to one side. Automatic methods cannot make breakthroughs, as this is undecidable and requires human ingenuity. Automatic methods like search can, however, optimally leverage a given type of attack or defence. If the optimisation defeats the opponent’s current capabilities, the disruption forces them to seek a breakthrough. Optimisation can end the cat and mouse incremental tweaks to existing detection and evasion methods, forcing both sides into a more expensive and creative search for disruptive techniques, slowing the race and taking away the structural advantage black hats currently enjoy.

The concept is to combine program analysis, search algorithms, and machine learning. These algorithms can artificially force evolution and coevolution in the malware detection arms race. We call this hothousing and use the word hothouse to mean a particular set of experiments that investigate the effect of learning algorithms on a given set of detection and evasion techniques. This idea about hothousing the arms race is related to game theory [11]. Zhou et al. described the connection between the arms race and adversarial machine learning as zero-sun game, where the benefits of the adversary are opposite to those from the learner. We designed hothousing as a sequential game, where each agent plays its strategy before the next. More precisely, hothousing is a form of Stackelberg game [12], consisting on a leader agent, in this case the adversary and a follower which responds, in this case the detection system.

The arms race in terms of disk resident executables on desktop systems can be understood as a coevolution between the anti-virus engines and the malware. Malware aims to execute its payload avoiding the detection while anti-viruses aim to detect and eliminate malware. This detection-evasion scenario evolves in two directions: the black hats design new concealment strategies based on packing, polymorphism and metamorphism, and anti-virus companies define new methods to increment their detection rate. This work introduces itself in this scenario to hothouse VirusTotal with EEE [2], the evolutionary packer. VirusTotal contains different anti-virus engines and provides detections from all the different engines. However, we have no knowledge about the detection features, data or algorithms that the anti-viruses are using.

Our *Research Hypothesis* is: EEE will generate malware with better concealment abilities, forcing VirusTotal to improve the detection performance. Evolving EEE will also make VirusTotal evolve and improve the detection of the individual antivirus engines.

Several works are based on the assumption of knowing these three factors or combinations of them [13]. According to Srndic and Laskov, our scenario is one of the unexplored areas of adversarial machine learning [14]. We also include inside this area the concept of detection system coevolution, i.e., the uncontrolled evolution of the detection system to the adversary, which, from the knowledge of the authors, is a completely novel area. During the iterations of this coevolution, EEE parts from a detected malicious instance, modifies the program shape to create a variant and uses this variant to attack the anti-virus. As the anti-viruses respond to the attacks, we will improve the skills of the packer during the hothousing process, to understand how the other side responds and evolves.

## 3. EEE: The Evolutionary Packer

In previous work [2], we considered a relatively unexplored malware detection technique based on information theory. This approach, called EnTS, aims to distinguish disk resident malware from benign-ware based on different entropy pattern. The insight was that polymorphic and metamorphic payload hiding generate regions in a malware binary with high entropy relative to benign-ware. This family of detection techniques extracts entropy signatures from binaries and uses them to distinguish malware from benign-ware. To hothouse EnTS, we built EEE, an adversarial packer that controls binaries’ entropy signature. EEE easily defeated EnTS and other state of the art detectors based on machine learning by using incremental moves. This section presents the evolution from UPX to EEE [2], summarized in Table 1, and the design and work-flow of the evolutionary packer at the point where we broke off its coevolution with VirusTotal.

### 3.1. Evolving UPX to EEE

Normally, we would report only on the final version of the packer, but since we found ourselves in an arms race with VirusTotal, we must mention the five versions of UPX and EEE used in the experiments (Table 1). Our first step was the application of the original UPX, use of which corresponds to point B in Figure 1. After, we create the first attempt of MUPX-I, including XOR encryption after the compression process. It corresponds to point C in Figure 1. MUPX-II updated with controlled entropy regions (CERs) that have fixed length, fixed (low) entropy, and start positions sampled from a uniform distribution. Experimental results using MUPX-II correspond to points D and E in Figure 1. MUPX-III updated with a more sophisticated ability to vary the configurations of its CERs. It uses CER descriptors and samples the initial positions from one of a family of Gaussian distributions. Experiments using MUPX-III correspond to points F and G in Figure 1. EEE added the genetic search algorithm that uses detection frequency as a fitness function. It also includes the protections described in Section 3.2. Experiments with EEE correspond to point H in Figure 1.

### 3.2. EEE Protections

Since unpacking could remove the CERs that EEE inserts into the packed binary, we needed to take measures to force the AV engines to consider the packed mode as much as possible by resisting dynamic analysis. This resistance took two forms: encryption and the addition of an execution delay.

UPX is sufficiently widespread and well-known that anti-virus engines can detect signatures in its compressed code. In order to forestall this, we added simple XOR encryption. It is important that the encryption does not modify entropy. If the encryption significantly increases a binary’s entropy, the binary will be less compressible, reducing the space available for CERs to occupy. XOR encryption modifies neither the size nor the entropy of a file, since, when a byte key is used, XOR permutates the file bytes. The decryption code is packed into the stub along with the key. We provide the stub with the virtual memory positions of the encrypted sections to ensure it can find them in real time. The decryption is performed after the uncompression and before reconstructing the PE imports.

Protection against dynamic analysis is technically challenging. As an initial, simple measure against dynamic analysis for EEE, we introduced a (mutable) time delay before the beginning of the unpacking process, controlled by the input parameters (and therefore by the genetic algorithm).

The chief focus of our modifications to UPX to produce EEE is the stub. The modifications increase the size of the stub and make it different from the stub of the original UPX, and hence a potential attack surface for detecting EEE. To resist the detection of the stub as a signature for the packer long enough to do the experiments, we divided all the code that is not original to UPX into 12 small parts. We generate 4 different semantically equivalent versions for each of these 12 parts. Selecting and combining them oligomorphically as part of the genetic algorithm allows the packer to generate around 10,000 different versions of the stub.

### 3.3. EEE Final Version

**Figure 2:**
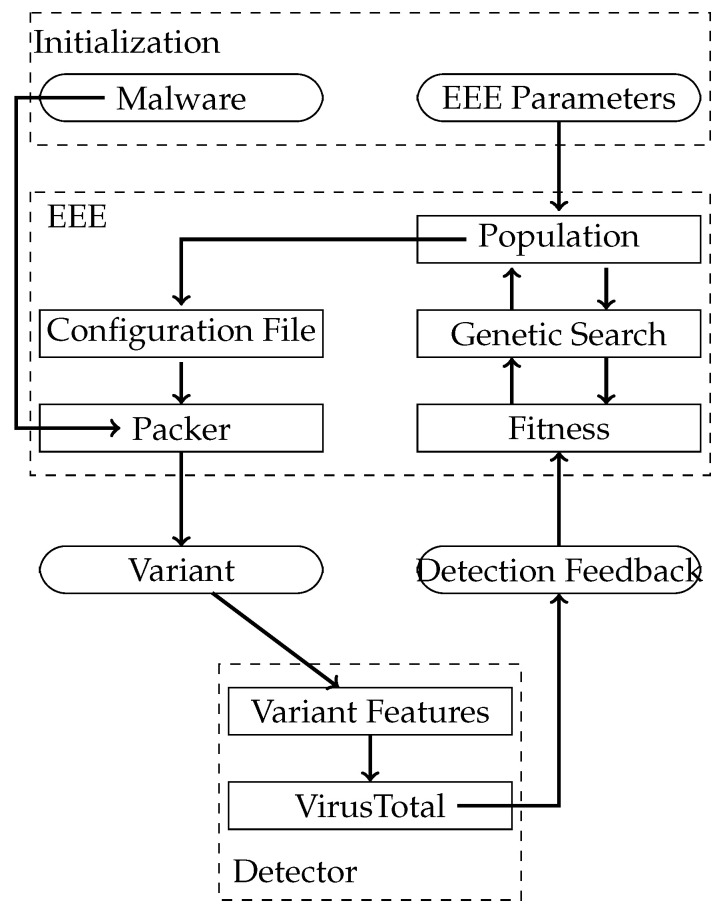
The architecture of EEE, the Evolutionary Packer, showing the initialization of the packer and the GA at top, the interactions among the components of EEE and the interaction with the malware detector at bottom.

EEE architecture is divided into a packer that incorporates all the protections attributes mentioned above and a genetic algorithm that decides how these protections are applied depending on the response of VirusTotal (Figure 2). In the figure, we can see how the whole process works from a malware instance to the generation of multiple malware variants. EEE starts with the original malware and a set of parameters that determine different aspects, such as the position of the CERs, the delay, and the version of the stub. This goes into EEE that generates a population configurations related to these parameters. This population, combined with the original malware, creates a set of variants that are sent to the detection technique, in this case VirusTotal. VirusTotal provides some feedback, used as a fitness function and EEE uses this feedback to readjust the configurations and create new variants. This process goes on until the detection rate for the variants is close to zero.

**Figure 3:**
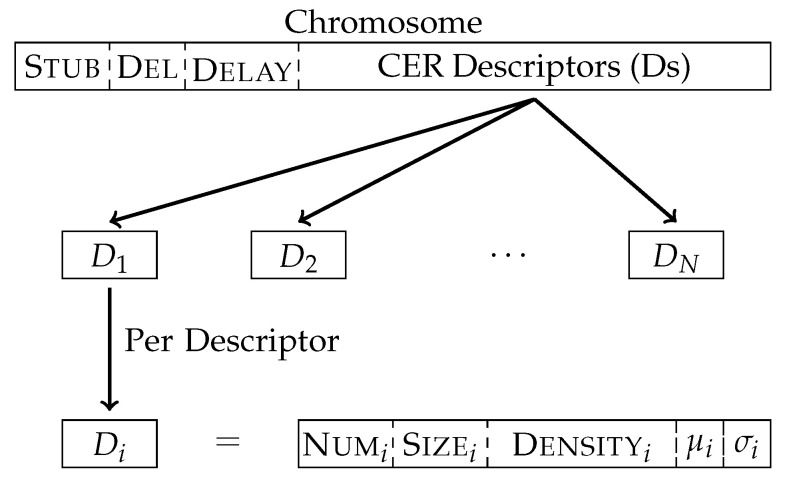
EEE Chromosome scheme. A chomosome is a vector where the first coordinates correspond to the stub, followed by the delimiters and the delay; then follows the types of CER, where the information for each descriptor is the number of CERs for that descriptor, the Size, the Density, and the parameters for the position distribution.

In order to optimise the effect of injecting CERs, selecting a stub and the time delay, EEE uses a genetic algorithm that manages the size, density and position of these CERs within the binary. The population of the algorithm are different possible parametrizations, represented as a vector of real numbers (Figure 3). The initial population sets these values uniformly at random.

Every element of the population is a chromosome. They define the search space used to generate variants. Figure 3 shows the components of a chromosome: Stub, delimiter (Del), delay, and CER descriptors. CER descriptors describe the properties of different groups of CERs. There is a limited number of descriptors to bound the search space while still allowing sufficient variation on CERs inside a program. For each descriptor, the search optimizes: Density, Size, position (μ, σ) and number of regions (Num). Stub identifies an oligomorphic version of the stub.

The algorithm makes decisions according to the feedback provided by the detection system, which works as a fitness function. For each configuration, or chromosome, EEE generates a variant and submits it to VirusTotal to get feedback. This feedback is the percentage of VirusTotal AV engines that detect each submitted variant. This percentage is the fitness value. To select individuals for survival, it retains some elite individuals, then applies a tournament operator to chose individuals for reproduction via crossover. Finally, a mutation operator is applied to all individuals and the search continues, creating new variants from each individual of the new generation. If, after a fixed number of generations, the algorithm finds no improvements in the detection probability, it stops. The fittest variant (i.e., the one with the lowest detection probability) is chosen as the best individual of the search.

Although UPX, being a packer, preserves the prepacking program semantics after packing and unpacking, our modifications to UPX always ran the risk that they would break this semantic invariance. In response we introduced a check that the invariance held that generates reports from the software execution. This system is called Zero Wine Tryouts [15]. We generated reports for both the original binary and the packed one and compare their traces. We also compare memory dumps after unpacking. If the traces and memory are identical, after the EEE variant finishes its unpacking process, we consider that semantics of a program to be unchanged by EEE-packing.

For more details about the algorithm, please refer to [2] (EEE is open source and available at https://github.com/hdg7/EEE (accessed on 20 March 2021).

## 4. Experiments

EEE uses search to adapt to its adversaries. Here, we hothouse it against VirusTotal to learn how well entropy-aware evasion, which is an incremental and automatic move, works against the state of the art anti-viruses. Our main research questions are:

- How much does this modified version of UPX reduce the detection rate for real antivirus? (Section 4.1)
- How quickly does VirusTotal learn from the programs submitted to it? As we detect false positives, we also asked, How quickly does VirusTotal recover from false positives? (Section 4.2)
- How much does automated search using a genetic algorithm allow EEE to reduce VirusTotal’s detection rate? (Section 4.3)
- Which antivirus tools are more resistant and what are they detecting? (Section 4.4)

**Figure 4:**
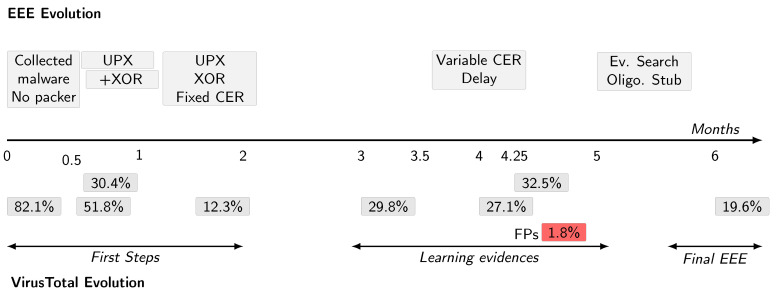
Chronology of VirusTotal and EEE evolution (time in months).

The aim of all these questions is to learn the arm race evasion form the black hats perspective. Our central finding shows unsuprisingly that AV focuses on the packer loader (stub). When we masked it with random bits and restirct AVs to consider the rest of the binary, EEE utterly defeats current AVs (Section 4.4). Figure 4 shows the timeline of our experiment pictorially. We begin with our experimental setup and corpus.

**Experimental Setup**. We ran our experiments on a cluster of 6 computers equipped with 24 cores and 128 Gb of RAM memory each. The state of the art in malware detection is VirusTotal, a web service comprising 56 anti-virus tools. To manage its traffic, VirusTotal throttles to 4 submissions per minute and originating IP address. Our six machines submitted candidate malware to VirusTotal. To accelerate queries, we issued requests to VirusTotal from multiple accounts on different IPs in parallel. Unusually, we do not evaluate EEE on train and test data, because VirusTotal, at a rate that we quantify below, learns and changes its response, violating the assumption on which machine learning rests that train and test share the same distribution [16]. Unless stated otherwise, experimental outcomes are reported as a percentage detection rate. The detection rate should be interpreted as follows: for each individual variant we calculate the percentage of VirusTotal engines that detect that variant. Then we report the median rate for all variants submitted to VirusTotal during the experiment.

**The Dataset**. We collected malware from a public malware database named VirusShare (http://virusshare.com (accessed on 31 January 2016) ), from June 2015 to January 2016. The corpus has 4677 PE32 (Windows binary executables for 32 bits) malware compatible with UPX. Table 2 shows statistics about the size of our dataset in KB.

### 4.1. Initial Steps

Our first prototype, which covers the first two months of Figure 4, adds XOR encryption and fixed CERs to UPX. To measure the contribution of these features we first ask: “How much does this modified version of UPX reduce the detection rate for real antivirus?”.

For this experiment, we construct and submit four malware variants to VirusTotal: (1) the original malware, (2) packed with UPX, (3) packed with MUPX-I, and (4) packed with MUPX-II. We performed this experiment on 200 malware.

**Figure 5:**
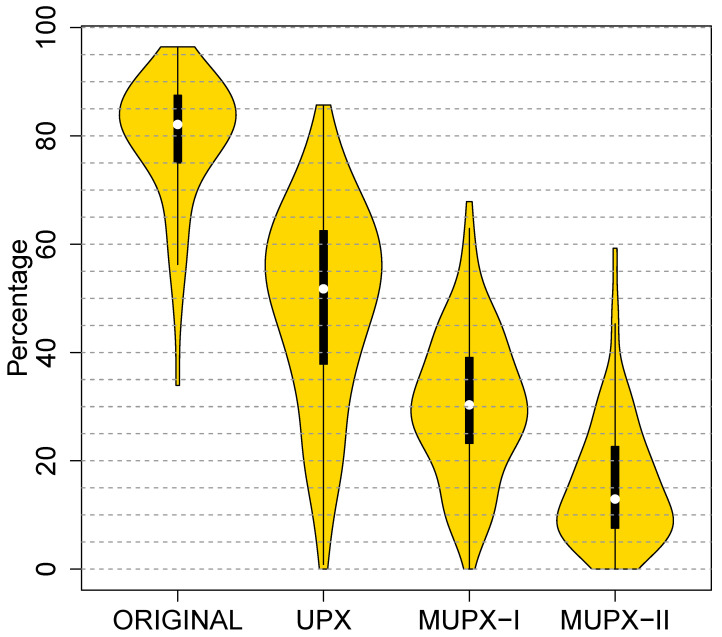
Initial performance of EEE. From left to right: detection on the original malware; detection after applying UPX; detection after applying MUPX-I; and detection after applying MUPX-II.

Figure 5 shows how the detection drops from left to right. The figure contains four violin plots, which combine a box plot with a kernel density plot, a nonparameteric estimation of a probability density functon [17]. Intuitively, the width shows where most events occur. The leftmost violin plot shows VirusTotal’s detection rate dropping from 81.2% on the “Original” malware to 51.8% for “UPX” packed malware. Since virus-checkers can recognize UPX-packed binaries and use UPX to analyse their unpacked contents, the first feature we added to UPX extends UPX with XOR encryption to produce MUPX-I. Figure 5 shows that the addition of “XOR” reduces the median detection rate to 30.4%. On the MUPX-I experiment, VirusTotal’s output labelled most of our variants as Yoda Protector [18] variants. Investigation revealed that Yoda Protector originally used XOR encryption, so many detectors handle it which probably explains why this rate does not fall further. Finally, we added CERs to MUPX-I to produce MUPX-II. (the rightmost violin plot in Figure 5). CERs significantly reduced the detection to a median of 12.3%. However, this initial success did not last long.

### 4.2. VirusTotal Is a Fast Learner

To drive VirusTotal’s detection rate still lower, we hypothesised that some of VirusTotal’s constituent antivirus tools were defeating MUPX-II in two ways: by detecting (1) the size or anomalous entropy of the CERs that MUPX-II injects or (2) via removal of CERs and XOR decryption during reconstruction of the binary under dynamic analysis. To impede dynamic analysis, we built a new version of MUPX, MUPX-III, that implemented the common tactic of delaying action; to prevent the incorporation of CERs into a malware signature, MUPX-III had the ability to vary their size and entropy. This covers the months 3 and 4 of Figure 4. When we first tested these features, we were surprised to see our performance degrade from 12.3% to 29.8%. We expected VirusTotal to improve—indeed our aim is to study the malware arms race itself—just not so quickly. Thus, this experiment asks “How quickly does VirusTotal learn from the programs submitted to it?”

To ensure that VirusTotal did not simply over-fit the initial malware corpus, we resampled the data. The new corpus contained 500 malware. MUPX-III’s parameters were delay, delimiter, and for CER descriptor, their number, length, entropy, and position. We fixed delimiter length to 8, then set delay uniformly over [0,300] seconds, delimiter characters uniformly over [0,255], the number of CERs per descriptor over [1,20], and the rest uniformly over [0,1]. We used these settings to create 20,000 variants per day (40 per malware and day).

**Figure 6:**
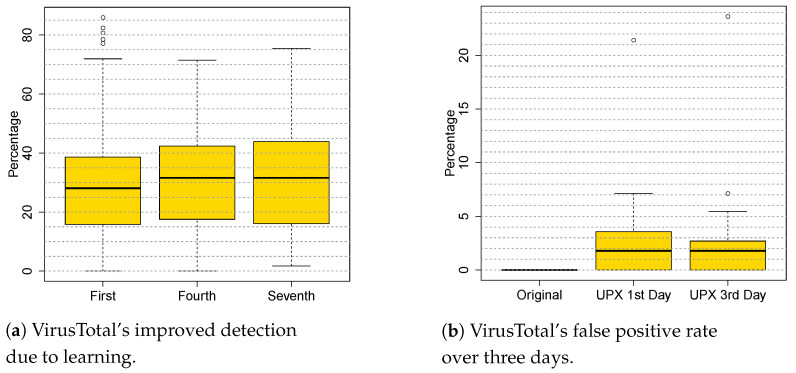
Learning and unlearning in VirusTotal.

Figure 6 (left) shows VirusTotal’s performance on this corpus, queried three time over a week, each query separated by three days. VirusTotal detects only 27.15% of the variants on the first day, then only three days later improves by 5 points to 32.55% on the fourth day. This shows the speed at which VirusTotal learns. The queries on the 7th day detection did not continue to increase. Looking at the data in detail, we discovered that three antivirus tools account for all of the improvement between the first and second queries (they improved 21.6, 45.1 and 13.0 points, respectively), and no additional anti-virus tools joined them at the third query. In short, VirusTotal learned from EEE in just three days.

VirusTotal’s improved detection of EEE packed malware had a side-effect: it caused anti-virus software to incorrectly classify UPX-packed benign-ware as malware. We discovered this fact when the anti-virus software running on the PC of one of the authors flagged UPX-packed benign-ware. VirusTotal had paid the price of incurring false positives to detect EEE-packed malware. Users are intolerant of false positives, so we asked: “How quickly does VirusTotal recover from false positives?”.

Under the assumption that VirusTotal had learned an incorrect signature, we collected 200 samples of benign-ware from download.com (accessed on 1 February 2016). First, we submitted them to VirusTotal to guarantee that none of them was considered malware, then we packed them with UPX and immediately resubmitted them before VirusTotal could consider them malware. Figure 6 (right) shows that the median malware classification rate was 2%, with the 3rd quartile at 7%. Two days later, we resubmitted the same set of UPX-packed benign variants. The median detection rate did not change significantly, but 3rd quartile dropped from 7% to 5%: some of the anti-virus tools in VirusTotal had removed the signature of these variants in 2 days.

### 4.3. Automatically Adapting to VirusTotal

Since, as we have shown, VirusTotal rapidly adapts to its inputs, our final modification of UPX updated MUPX-III to EEE, a version that incorporates an evolutionary algorithm to automatically learn from VirusTotal’s response. Together, VirusTotal and EEE reprise the malware arms race as the coevolutionary interplay of white and black hats. This last part covers the last two months of Figure 4. In addition to enabling EEE to evolve automatically, we hypothesised that VirusTotal was detecting the stub that MUPX-III embeds in its output, so in EEE we protected the stub oligomorphically, as previously discussed (Section 3) and made this protection evolvable by the genetic algorithm. This section measures how well EEE’s learning version performs against VirusTotal and asks “How much does automated search using a genetic algorithm allow EEE to reduce VirusTotal’s detection rate?”.

For this experiment, we re-sampled and chose a corpus of 1000 malware. We were not able to optimize the configuration of the genetic algorithm, since any parameter evaluation experiments would influence detection rates for VirusTotal and affect what followed, providing no useful conclusions about parameter configurations. Therefore, we selected them by trying to keep a compromise between diversity, in terms of population and reproduction, and learning in terms of generations and crossover.

We configured the evolutionary algorithm to contain 50 individuals. In each generation, we select 30 individuals for reproduction. We passed the ten fittest individuals of each generation to the next generation. The crossover probability was 0.8; the mutation probability was 0.1. The algorithm terminated either after 20 generations or once fitness failed to improve for 5 consecutive generations. The aim was to reduce the number of queries to VirusTotal. The parameter search space is the same as that used in Section 4.2, except the part for the stub, which we introduced in this experiment. The search combines stub components under the constraints detailed in Section 3. With these parameters, EEE produces at most 50 (individuals) ×20 (generations) =1000 variants per malware.

**Figure 7:**
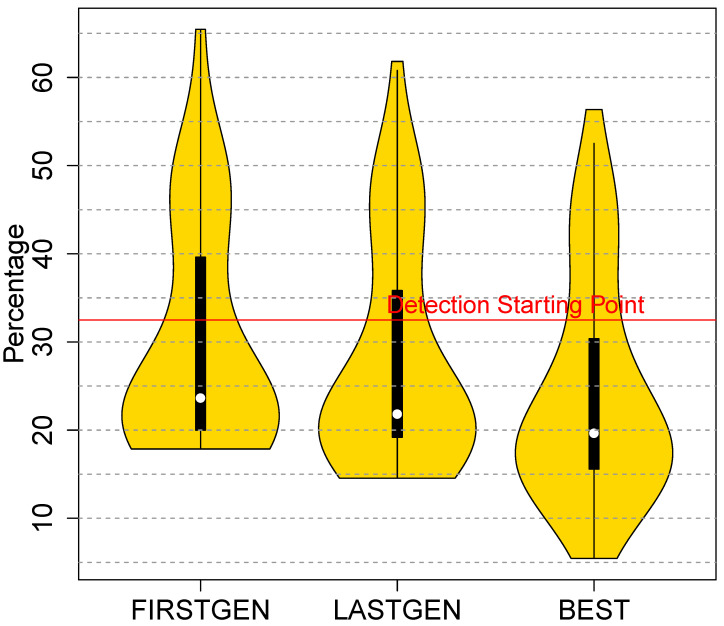
VirusTotal’s detection rate as EEE evolves to resist it, where FirstGen is EEE’s first generation, LastGen is the last generation, and Best contains the best individuals in the last generation.

Figure 7 compares VirusTotal’s detection rate across 3 collections of variants. The first (FirstGen) is the first generation and is equivalent to a random search due to initialization; the second (LastGen) is aggregate performance of the last generation; the third (Best) contains the most evasive individuals for each malware. Figure 7 shows a drop in the median detection rate from 21.8% to 19.6%.

In fact, the violin plots show that the distributions are becoming narrower with the interquartile range shrinking from [30,90] to [5,55] and more skewed. While their medians are close, FirstGen and LastGen represent different distributions. To confirm it, we applied the Wilcoxon statistical test, normally used for non-normal distributions, with a *p*-value limit of 0.05 for the null hypothesis. The *p*-value comparing these two distributions is $9.8\cdot 10^{-3}$. This is lower than 0.05, hence, they are significantly different. For instance, FirstGen’s interquartile range is [18,65] and LastGen’s is [15,60]. This effect is more striking when we consider the most evasive individuals EEE produces across all generations for each malware in the corpus. Black hats using EEE could pick out and store these individuals. VirusTotal’s performance on Best drops to a median detection rate of 19.6%. We also applied the Wilcoxon test to comparisons of Best with FirstGen, and Best with LastGen. The *p*-values were $2.2\cdot 10^{-16}$ and $2.1\cdot 10^{-12}$, respectively, therefore, they are also significantly different distributions. As a sanity check, we compared VirusTotal’s performance on Best with its performance on the corpus after UPX packing and found that VirusTotal’s detection rate was 20 percentage points higher on the UPX-based variants. The oligomorphic hiding of the stub parameters was evolvable via the genetic algorithm so the introduction of this stub protection was folded into the automated evolution. As the results show, employing the new capabilities of EEE effectively and substantially reduces VirusTotal’s detection rate, dropping it from 32.5% before applying EEE to 19.6% afterwards.

### 4.4. VirusTotal’s Resilience in Depth

In the previous experiment, EEE did not drive VirusTotal’s detection rate to zero. Clearly, some anti-virus tools are defeating EEE. Which ones?

**Figure 8:**
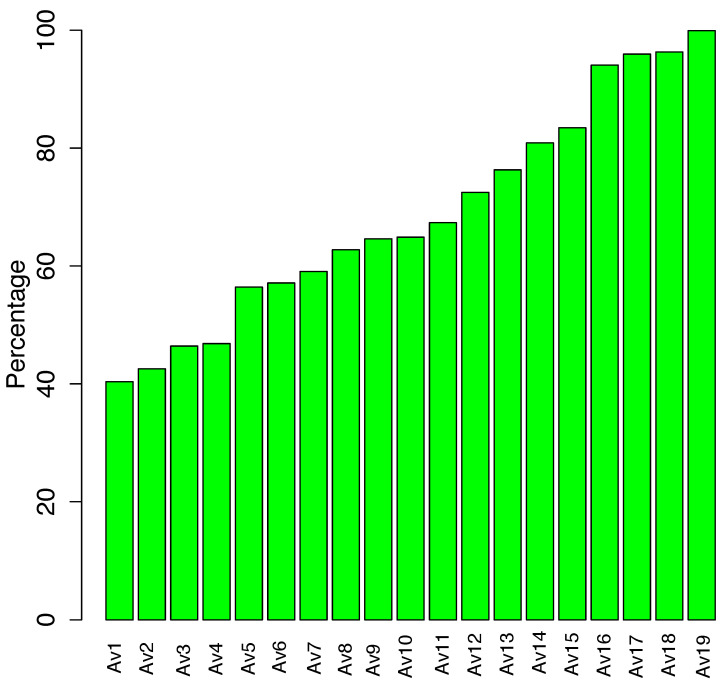
VirusTotal’s anti-virus engines that perform best against EEE.

Figure 8 shows the detection rates of the most EEE-resistant anti-virus engines in VirusTotal over all the variants generated during EEE’s variant search. This figure anonymises the anti-virus engines’ names, following the terms and conditions of VirusTotal. Four of VirusTotal’s 56 anti-viru —Av16, Av17, Av18 and Av19—correctly classified EEE variants in more than 90% of the trials. How are these successful anti-virus tools detecting EEE-protected variants?

We studied the sections of the packed files that EEE generated and their similarities. The first section, UPX0, was identical in all cases, because it is physically empty, since it is a virtual section into which the binary is unpacked at execution time [19]. UPX1 contains the packed code and the stub. The packed code was quite dissimilar across variants. The stub, however, did exhibit similarities across the variants. Inside it, the CER and the XOR handlers provide two attack surfaces. Because we induced false positive in VirusTotal in our learning experiments (Section 4.2), we hypothesised that some engines had found signatures inside the UPX stub, so we also considered the rest of the stub as an attack surface. Finally, we identified two attack surfaces in section UPX2: the imports list and the overlay of the program. Executables use the overlay section, which is not read into memory, to read themselves as a file. UPX keeps this section untouched to preserve the execution semantics. We selected 1000 of the most easily detected variants EEE produced and eliminated the potential detection areas (using the dd command and setting their bytes to 0). We then submitted them to VirusTotal.

**Figure 9:**
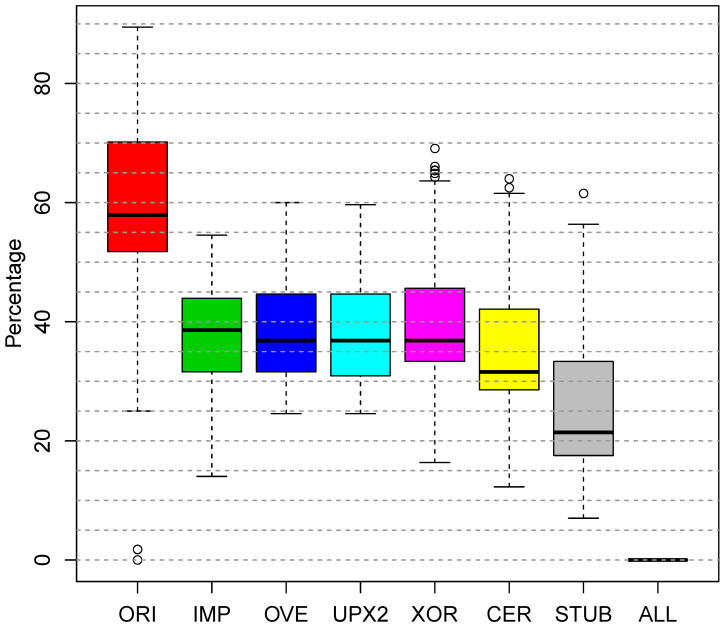
Detection boxplots for highly detected EEE-produced variants produced by zeroing specific parts of them: the leftmost boxplot is the original variant; in the middle are variants produced by zeroing each named region in isolation; the rightmost boxplot zeros all the named regions.

Figure 9 shows how the detection rate varied per modification. On the left, it shows VirusTotal’s detection rate on the unaltered variants Ori; this serves as the baseline. The rest of the boxplots show the detection after zeroing: the CER and XOR handlers, the entire stub Stub, the imports IMP, the overlay (OVE), UPX2 section and All of these six areas. The relationships among the six areas follow:

$$\begin{array}{cc} \mathrm{CER}\cap \mathrm{XOR} & =Ø\\ \mathrm{CER}\cup \mathrm{XOR} & \subset S_{TUB}\\ \mathrm{OVE}\cap \mathrm{IMP} & =Ø\\ \mathrm{OVE}\cup \mathrm{IMP} & \subset \mathrm{UPX}2\\ \mathrm{UPX}2\cap S_{TUB} & =Ø\\ \mathrm{UPX}2\cup S_{TUB} & =A_{LL} \end{array}$$

Zeroing the IMP, OVE, UPX2, and XOR all had roughly the same effect, dropping VirusTotal’s detection rate from 57.1% into [35.9,38.7]%. The CER handler was the most detected area of the stub. Its zeroing dropped VirusTotal’s detection rate to 32.1%. We added oligomorphism to EEE to protect the CER and XOR handlers, as discussed in Section 3. Clearly, these handlers need stronger protection. Eliminating the stub entirely dropped the detection rate to 20.5% by restricting VirusTotal to considering only UPX2. Zeroing all six areas (All) dropped the detection to 0. These results show that the EEE-resistant anti-virus engines are concentrating on the stub and ignoring the CERs, at this stage of the arms race with EEE.

From the 56 engines, only 46 generated detections during the whole experiment, the rest activate less often. 39 engines started with a detection rate over 60% of detection and 34 over 80%. After the the CERs improvement only 3 of them stayed over the 60% threshold, these 3 are Av15 (62.5%), Av2 (72.7%) and Av19 (73.5%). During the month when all the tools were learning, and before the resubmission of the variants, 7 engines reduced their signatures, reducing, as a side effect, their detection abilities. 39 engines incremented the number of signatures during this period of time, making their evasion harder. The second improvement of EEE was relevant against 20 engines, the rest stayed or improved their detection. Finally, the introduction of the evolutionary learning process was useful against 36 but 5 of them start to become resistant. At the end of the experiment, 25 engines were in range [0,20]%, 14 in [20,40]%, 3 in [40,60]% and 4 between [60,65]%.

**Figure 10:**
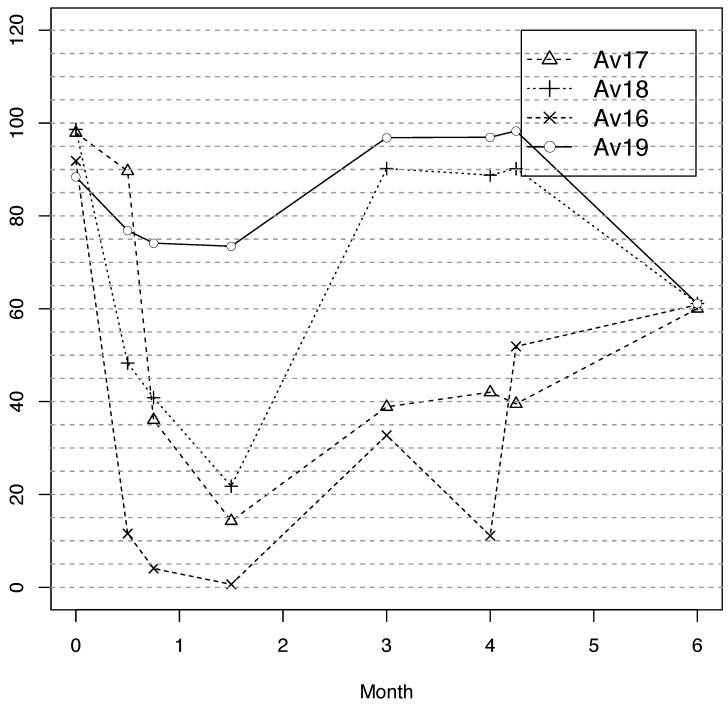
Detection rates over time for the anti-virus against EEE.

Completing the previous information, we back-tracked the detection of the four top anti-virus during the whole period (those in the [60,65] range). Figure 10 measures detection percentage in terms of the number of variants detected (unlike all the other experiments in which detection percentage means percentage of VirusTotal AV tools) and shows that all of them were affected by the introduction of XOR and CERs, but Av19. Av18 and Av19 became the strongest techniques during the month and a half when UPX became MUPX-II was improved for the first time, achieving detection rates over 85%. Av16 and Av17 became stronger during this period, but not as strong as Av18 and Av19. The version with variable CERs did not affect Av17, Av18 and Av19 but Av16 dropped from 31.1% to 10.9% detection rate. However, in seven days Av16 improved its detection rate to 52.3%. The best variants of EEE combined with the search dropped the detection for Av18 and Av19 to the range [60,65]%, but Av16 and Av17 improved their detection to 60.0% and 60.9%, respectively. The tendency of all the anti-virus detecting the same percentage of malware at this stage suggested that they are sharing a specific signature that, following the previous experiments, was part of UPX2 or the Stub. Moreover, there were two main tendencies in the detection evolution: Av16-Av17 and Av18-Av19 tendencies. This suggests that these tools were sharing information.

## 5. Related Work

This section summarizes work that leverages adversarial machine learning in the arms race coevolution and entropy-based methods in malware detection.

### 5.1. Coevolutionary and Adversary Learning Models

The arms race is constantly evolving. Black hats discover new vulnerabilities almost every day that white hats aim to prevent. This clearly defines a coevolution in both sides of cybersecurity as Somayaji described in [5]. Although Somayaji never formalized the phenomenon, Guerra et al. [9] made strong efforts on understanding it from both sides, in the context of spam filters for e-mails. Their study, corresponding to 12 years of an spam filter evolution, shows clear evidence on how both, the spammers as black hats and the filters as white hats, were learning from each other. However, the authors did not introduce themselves in this arms race, as we have done with EEE. In our case, we did not (and were not in a position to) directly access different versions of VirusTotal anti-virus tools, as Guerra et al. did with spam filters. Moreover, as a consequence of the anti-virus learning process, we were not able to repeat the experimental setup of the experiments. We were limited by working in a real-time scenario, while Guerra et al. were working offline.

Some tools like EvadeML [4] or IagoDroid [1] work in a similar direction to EEE, as they use the detection probability as a guidance for the quality of their variants. However, in these cases there is no evolution on the learner’s side, after the attack. In adversarial machine learning there are some examples where the learner aims to respond to the attack. These examples are Globerson and Roweis [20] that manipulate features from samples to attack a Support Vector Machine classifier or Zhou et al. [21] that concretely define attack strategies against a Support Vector Machine. Similar adversarial machine learning scenarios are the work of Kantarcioglu et al. [22], where the classifier adapts its cut-off to the attacks in order to find an equilibrium between the attacker and the detection, and the work of Lui and Chawla [23], where they apply the Nash equilibrium between the attacker and the classifier.

Our step into the current arms race required a deeper understanding of learning methods, as they are currently the state of the art for both dynamic and static analysis [24]. This motivated us to apply adversarial machine learning, a field whose aim is to exploit vulnerabilities in machine learning models [25]. In our original work of EEE [2], we attacked machine learning algorithms using CERs, and we demonstrated the effectiveness of the approach defeating algorithms that can reach more than a 95% of accuracy detecting malware. Although this version of the algorithm was effective against machine learning, it could not deal with anti-virus as it leaked clear signatures. For that reasons we included dynamic and static analysis protectors, as described in Section 3.2. With these protectors, we were able to introduce EEE in the malware arms race against VirusTotal.

VirusTotal is indeed an interesting attack surface for black hats, as it contains several AV engines and it is in constant development. In the literature, there are several works leveraging VirusTotal as a comparison framework [26] or as ground truth for malware detection [27]. An interesting application of VirusTotal is AVClass, a tool whose aim is to identify the ground truth on the malware family classification problem by averaging the opinion of the different engines of VirusTotal [28]. There are also tools like MimickAV [29] that prove the predictability of VirusTotal and auditing studies that show how obfuscation changes the outcome of anti-viruses [30]. Nevertheless, from our current knowledge, no author has used it to study the malware arms race coevolution.

As adversaries of VirusTotal, we face a difficult scenario because we do not have any information about the strategies and features that the engines are using to detect malware. According of the taxonomy of evasion scenarios introduced by Srndic and Laskov [14], in this scenario, the adversary needs to deduce features by observation, for this reason we introduced our genetic evolution, in order to discover those features that make the detector vulnerable. Perturbation methods were originally succeded against spam detectors [31], where different alterations on emails defeated filters based on machine learning. Modern works, like Xu et al. [4], show the effectiveness on these alterations on malware misclassification, in their case, applied to well-knonw PDF malware detectors [32,33]. In contrast to evadeML, EEE knows nothing about the training data or the anti-virus features it attempts to attack, and it had no knowledge of nor any direct access to the workings of the anti-virus tools in VirusTotal. Moreover, our variants are based on Windows binary executable malware that uses protections against disassembly or reassemble, while evadeML manipulates PDF malware. Furthermore, UPX makes our tool compatible with several architectures, giving us the opportunity to adapt EEE to other platforms [19].

Adversarial machine learning is consolidating itself as one of the strongest fields in cybersecurity as it is a clear countermesuare to the machine learning peak [13]. It has studied concrete algorithms, as the work of Biggio et al. understanding the vulnerabilities of Support Vector Machines [34], and it is classfied into different generalization frameworks, depending on the adversary knowledge [35], and it is becoming a treath even for modern machine learning application as those based on manifolds [36] and those applied to the design of new technologies [37]. Other modern techniques on the area of adversarial machine learning for malware evasion has focused on attacking malware triage [1] of classification [2].

### 5.2. Entropy in Malware Detection

Our attack is based on an application of Shannon entropy [38], the fundamental concept in information theory, widely used in many sciences, from physics to biology and communications engineering. Several authors have employed this concept in the context of malware detection, using it as a measure of redundancy in bytes [39,40], program traces [41], program compression [42,43] and n-grams [44,45], among others. It is also a relevant feature for machine learning based malware detection [46,47,48].

Detection of malware via entropy measures is an obscure game. Entropy analysis activates detection flags in terms of compression and encryption, due to the altering of the entropy of binary files—usually by increasing it. Lyda and Hamrock used this approach to detect encrypted and packed malware [39]. However, their detection process was based on entropy ranges, which is easily manipulated. Jacob et al. used a similar methodology for detecting packed malware [49]. They produced four tests to determine whether the file is malware or not, starting by examining whether it is packed. This test measures the average entropy of the file and checks whether it is higher than a threshold. These two examples are strong motivation for attacking executable file entropy by reducing or manipulating it over a specific range.

A recent technique, named structural entropy, defines an entropy based similarity metric based on the Levenshtein distance and producing an entropy profile of the program [50]. This entropy profile consists of local entropy values for blocks of bytes of the binary. Bayse et al. leveraged this technique for malware detection [51]. Altering the entropy profiles at the file level would introduce noise into the detection, another motivation for introducing attacks on detection via controlled entropy regions.

## 6. Conclusions

We tracked and analysed the behaviour of VirusTotal malware detectors in a coevolutionary contest with EEE. Hothousing anti-virus engines accelerates the malware arms race, reducing black hats’ scope for leveraging incremental moves. After the hothousing experiments we conducted, evolutionary search algorithms reduced the median detection rate for the state of the art in malware detection by over a third. Over six months of experimentation, we witnessed VirusTotal’s learning behaviour; in particular, we saw it learn to detect EEE-packed malware via EEE’s loader (stub). Initially, our experiment caused VirusTotal to generate false positives on benign-ware packed with UPX; an error, from which VirusTotal recovered, after 2 days. At the end of the experimental run, only four of the 56 VirusTotal AV engines were effectively resisting EEE. Hothousing promises to automate evolution on both sides of the coevolutionary malware detection arms race, using detection and evasion techniques other than those based on entropy, so white hats can stay a step ahead of black hats.

## 7. Availability

EEE has been published and it is available in the following URL: https://github.com/hdg7/EEE (accessed on 20 March 2021). Use it only for academic and benign purposes.

## Figures and Tables

**Table 1:** Evolution from UPX to EEE and its attributes connecting it with the different versions presented in Figure 1.

Version	Compress	Encrypt	CERs	Search	Protect	Points
UPX	Yes	-	-	-	-	B
MUPX-I	Yes	XOR	-	-	-	C
MUPX-II	Yes	XOR	Fixed	-	-	D-E
MUPX-III	Yes	XOR	Variable	Random	Delay	F-G
EEE	Yes	XOR	Variable	Evolutionary	Delay & Oli	H

**Table 2:** Size distribution in KB of the malicious software corpus.

	Median	Mean	Min	Max
Malware	392	717	4	17,972

## Data Availability

Data is contained within the article.
